# Family history of breast cancer increases the risk of prostate cancer: results from the EPICAP study

**DOI:** 10.18632/oncotarget.25320

**Published:** 2018-05-04

**Authors:** Pierre-Jean Lamy, Brigitte Trétarre, Xavier Rebillard, Marie Sanchez, Sylvie Cénée, Florence Ménégaux

**Affiliations:** ^1^ Service Urologie, Clinique Beau Soleil, Montpellier, France; ^2^ Institut d’Analyse Génomique-Imagenome, Labosud, Montpellier, France; ^3^ Registre des Tumeurs de l’Hérault, ICM, Montpellier, France; ^4^ Université Paris-Saclay, Université Paris-Sud, CESP (Center for Research in Epidemiology and Population Health), Inserm, Team Cancer and Environment, Villejuif, France

**Keywords:** family history of cancer, epidemiology, prostate cancer, breast cancer, Gleason score

## Abstract

**Introduction:**

Familial aggregation is now well established with an increased risk of prostate cancer in patients with a family history of prostate cancer in first degree relatives. The aim of this paper was to investigate the role of family history of cancer in first degree relatives in prostate cancer risk.

**Results:**

As expected, a family history of prostate cancer in first-degree relatives was more frequent in cases than in controls (OR 3.10, 95% CI 2.32–4.15). A family history of early BCa (before age 50) in first-degree relatives was more frequent in cases than in controls (OR 1.79, 95% CI 1.09–2.94) with higher risk of aggressive PCa. The association was more pronounced for BCa in daughters (OR 15.26 95% CI 1.95–120).

**Conclusions:**

In summary, a family history of BCa in first degree relatives before age 50 may increases the risk of PCa with higher Gleason score. This finding could suggest a specific prostate surveillance and/or genetic counselling for men who present such familial history.

**Methods:**

EPIdemiological study of Prostate CAncer (EPICAP) is a population-based case-control study specifically designed to investigate the role of environmental and genetic factors in prostate cancer. Detailed information on family history of cancer in first degree relatives (parents, brothers and sisters, children) was collected as well as the age of occurrence and the localization of each cancer. Overall, 819 cases and 879 controls have been included.

## INTRODUCTION

In most Western countries, prostate cancer (PCa) is by far the most commonly diagnosed cancer in men, which represents, in France, an estimated 53,917 new cases and 8,685 deaths in 2011 [1]. Despite a relatively high morbidity and mortality (3rd leading cause of cancer death in France), only age, ethnicity and a family history of PCa are well-established risk factors of PCa, and except those factors, the aetiology of PCa remains largely unknown.

Familial aggregation is now well established with an increased risk of PCa in patients with a family history of PCa in the first-degree relatives [2–6]. This risk is even more important if the affected relative is a brother and if the number of relatives affected is large [7, 8]. Family studies have also shown that some PCas can be inherited as an autosomal dominant model and it has been estimated that PCa family, due to a rare gene with high penetrance, accounted for approximately 10% of all PCa [9] Recently, genome-wide association studies have identified at least 100 susceptibility loci associated with PCa [10]. Individually, they contribute to a small increase in PCa risk but taken together approximately 30% of the familial risk is due to such variants.

If it is known that prostate cancer can run in families, it is not known whether other cancers are frequent in such families. Recently, familial associations between PCa and other cancers has been studied in a Swedish cohort suggesting that breast, kidney, nervous system tumors and myeloma occur more often in families of PCa patients [11]. In that context of a possible familial risk, there is growing evidence for clustering of breast and prostate cancer and that breast cancer family history may also influence prostate cancer risk. [12–17].

The aim of this paper was to investigate the role of family history of cancer in first-degree relatives, based on data from the French EPICAP study.

## RESULTS

The characteristics of the study population are presented in Table 1. Age in 5-year groups, ethnic origin, educational level, smoking status and body mass index were similarly distributed between cases and controls (*p* = 0.14, *p* = 0.23, *p* = 0.37, *p* = 0.08, *p* = 0.91, respectively).

**Table 1:** Study population characteristics

	Cases *n* = 819 (%)	Controls *n* = 879 (%)	*p*-value^1^
**Gleason score at diagnosis^2^**			
<7	341 (41.6)	-	
7 (including 3 + 4)	282 (34.4)	-	
>7 (including 4 + 3)	183 (22.3)	-	
**Age (years)**			0.14
<55	48 (5.9)	59 (6.7)	
[55–60]	99 (12.1)	99 (11.3)	
[60–65]	217 (26.5)	201 (22.9)	
[65–70]	274 (33.5)	285 (32.4)	
≥70	181 (22.1)	235 (26.7)	
**Ethnic Origin**			0.23
Caucasian	795 (97.1)	859 (97.7)	
Others	24 (2.9)	20 (2.3)	
**Educational level**			0.37
Middle school	70 (8.5)	72 (8.2)	
Up to high school	376 (45.9)	436 (49.7)	
High school	113 (13.8)	110 (12.5)	
College	260 (31.7)	260 (29.6)	
**Body Mass Index (kg/m^2^)**			0.91
<25	297 (36.8)	316 (36.6)	
[25–30]	377 (46.7)	395 (45.8)	
≥30	134 (16.5)	152 (17.7)	
**Smoking status**			0.08
Never	240 (29.3)	246 (28.0)	
Former	455 (55.6)	476 (54.2)	
Curent	123 (15.0)	157 (17.9)	
**Number of first degree relatives**			0.026
2 to 5	289 (35.6)	263 (30.0)	
6 to 9	421 (51.8)	473 (54.1)	
≥10	103 (12.6)	139 (15.9)	

^1^Adjusted for age (except for age).

^2^Category « Gleason = 7 » includes subjects for whom the two most commonly represented grades in the tumor are not known, as well as those for which the two grades are 3 + 4. Category « Gleason > 7 » includes subjects for whom the sum of the two most frequently represented grades in the tumor analyzed is > 7 and those for which the two grades are 4 + 3.

As expected in the literature, a family history of PCa in first-degree relatives was more frequent in cases than in controls (OR 3.10, 95% CI 2.32–4.15) (Table 2). In addition, the risk was more pronounced according to the number of family history of PCa in first-degree relatives (*p* trend < 0.001) and for early PCa in relatives (less than 60 years at time of diagnostic), either in father or in brothers (OR 4.04, 95% CI 1.60–10.2, OR 5.91, 95% CI 2.66–13.1, respectively).

**Table 2:** Associations between family history of prostate cancer (PCa) and prostate cancer risk

	Cases *n* = 819 (48.2%)	Controls *n* = 879 (51.8%)	OR^1^ 95% CI^2^
**Family history of PCa in first degree relatives**			
No	549 (75.2)	723 (90.4)	1.00 reference
Yes	181 (24.8)	77 (9.6)	3.10 (2.32–4.15)
Cancer >60 years	126 (17.4)	64 (8.0)	2.57 (1.86–3.54)
Cancer <60 years	49 (6.8)	13 (1.6)	5.15 (2.75–9.62)
**Number of family history of PCa in first degree relatives**			
None	549 (75.2)	723 (90.4)	1.00 reference
1	159 (21.8)	70 (8.8)	2.98 (2.20–4.03)
≥2	22 (3.0)	7 (0.9)	4.44 (1.87–10.6)
			*p trend < 0.001*
**Family history of PCa in fathers**			
No	549 (75.2)	723 (90.4)	1.00 reference
Yes	127 (18.8)	57 (7.3)	2.85 (2.04–3.98)
Cancer >60 years	104 (15.5)	51 (6.5)	2.60 (1.82–3.71)
Cancer <60 years	19 (2.8)	6 (0.8)	4.04 (1.60–10.2)
**Family history of PCa in brothers**			
No	549 (75.2)	723 (90.4)	1.00 reference
Yes	65 (46.7)	27 (45.8)	3.79 (2.36–6.10)
Cancer >60 years	33 (16.5)	18 (17.7)	2.73 (1.51–4.91)
Cancer <60 years	30 (16.5)	9 (17.7)	5.91 (2.66–13.1)

^1^OR: Odds Ratios adjusted for age, ethnic origin and number of male relatives.

^2^95% CI: 95% Confidence Interval.

A family history of early BCa (before age 50) in first-degree relatives was more frequent in cases than in controls (OR 1.79, 95% CI 1.09–2.94) which was not observed for family history of BCa after 50 years (OR 0.88, 95% CI 0.61–1.27. (Table 3). The number of family history of BCa and a family history of BCa in sisters or in mothers, whatever the age at diagnosis of cancer, were not associated with the risk of PCa. However, BCa in daughters was significantly associated with higher risk of PCa (OR 15.26 95% CI 1.95–120), even though based on small numbers. Looking at the associations between family history of BCa and PCa risk according to family history of PCa, only BCa in daughers in family with no history of PCa are relevant (OR 14.6 95% CI 1.85–116) (Table 4).

**Table 3:** Associations between family history of breast cancer (BCa) and prostate cancer risk

	Cases *n* = 819 (%)	Controls *n* = 879 (%)	OR^1^ 95% CI^2^
**Family history of BCa in first degree relatives**			
No	647 (84.5)	711 (85.4)	1.00 reference
Yes	119 (15.5)	122 (14.6)	1.13 (0.84–1.52)
Cancer >50 years	63 (7.7)	80 (9.1)	0.88 (0.61–1.27)
Cancer <50 years	45 (5.5)	32 (3.6)	1.79 (1.09–2.94)
**Number of family history of BCa in first degree relatives**			
None	647 (84.5)	711 (85.4)	1.00 reference
1	109 (14.2)	110 (13.2)	1.11 (0.82–1.51)
≥2	10 (1.3)	12 (1.4)	1.38 (0.55–3.43)
			*p trend* = *0.36*
**Family history of BCa in mothers**			
No	647 (84.5)	711 (85.4)	1.00 reference
Yes	62 (8.7)	69 (8.8)	1.04 (0.71–1.52)
Cancer >50 years	42 (5.9)	53 (6.8)	0.95 (0.61–1.48)
Cancer <50 years	20 (2.8)	15 (1.9)	1.39 (0.67–2.87)
**Family history of BCa in sisters**			
No	647 (84.5)	711 (85.4)	1.00 reference
Yes	55 (7.8)	60 (7.8)	1.10 (0.72–1.68)
Cancer >50 years	25 (3.2)	32 (3.6)	0.87 (0.49–1.54)
Cancer <50 years	16 (2.0)	16 (1.8)	1.36 (0.65–2.85)
**Family history of BCa in daughters**			
No	647 (84.5)	711 (85.4)	1.00 reference
Yes	11 (1.7)	2 (0.3)	15.26 (1.95–120)

^1^OR: Odds Ratios adjusted for age, ethnic origin, number of first-degree female relatives and famili history of prostate cancer in first-degree relatives.

^2^95% CI: 95% Confidence Interval.

**Table 4:** Associations between family history of breast cancer and prostate cancer risk according to family history of prostate cancer

	No family history of prostate cancer			Family history of prostate cancer		
	**Cases** ***n* = 549 (%)**	**Controls** ***n* = 423 (%)**	**OR^1^ 95% CI^2^**	**Cases** ***n* = 181 (%)**	**Controls** ***n* = 77 (%)**	**OR^1^ 95% CI^2^**
**Family history of BCa in first degree relatives**						
No	448 (84.1)	595 (84.9)	1.00 reference	150 (85.7)	65 (89.0)	1.00 reference
Yes	85 (15.9)	106 (15.1)	1.10 (0.81–1.51)	25 (14.3)	8 (11.0)	1.39 (0.57–3.35)
**Family history of BCa in mothers**						
No	448 (84.1)	595 (84.9)	1.00 reference	150 (85.7)	65 (89.0)	1.00 reference
Yes	46 (9.3)	59 (9.0)	1.03 (0.68–1.55)	13 (8.0)	5 (7.1)	1.18 (0.39–3.58)
**Family history of BCa in sisters**						
No	448 (84.1)	595 (84.9)	1.00 reference	150 (85.7)	65 (89.0)	1.00 reference
Yes	36 (7.4)	52 (8.0)	1.03 (0.65–1.63)	13 (8.0)	4 (5.8)	1.6 (0.48–5.66)
**Family history of BCa in daughters**						
No	448 (84.1)	595 (84.9)	1.00 reference	150 (85.7)	65 (89.0)	1.00 reference
Yes	10 (2.2)	1 (0.2)	14.6 (1.85–116)	1 (0.7)	0 (0.0)	–

^1^OR: Odds Ratios adjusted for age, ethnic origin and number of first-degree female relatives.

^2^95% CI: 95% Confidence Interval.

A family history of BCa before age 50 in a first-degree relative was more specifically associated with aggressive PCa (OR 2.42 95% CI 1.19–4.90). In the sub-class of family history in daughters, the risk was higher for aggressive PCa than for low or intermediate PCa (OR 9.25 95% CI 1.66–51.6, OR 5.64 95% CI 1.16–27.5, respectively), even though the difference was not statistically different. (*p* = 0.42) (Table 5).

**Table 5:** Associations between family history of breast cancer and prostate cancer risk according to Gleason score

		Low or intermediate grade prostate cancer Gleason score ≤ 7 (3 + 4)		High grade prostate cancer Gleason score ≥ 7 (4 + 3)	
	**Controls** ***n* = 879 (%)**	**Cases** ***n* = 623 (%)**	**OR^1^ 95% CI^2^**	**Cases** ***n* = 183 (%)**	**OR^1^ 95% CI^2^**
**Family history of BCa in first degree relatives**					
No	711 (85.4)	498 (85.6)	1.00 reference	138 (80.2)	1.00 reference
Yes	122 (14.6)	84 (14.4)	1.04 (0.76–1.41)	34 (19.8)	1.42 (0.92–2.19)
Cancer >50 years	80 (9.1)	48 (7.7)	0.86 (0.57–1.29)	14 (7.7)	0.96 (0.52–1.77)
Cancer <50 years	32 (3.6)	32 (3.6)	1.66 (0.97–2.85)	13 (7.1)	2.42 (1.19–4.90)
**Number of family history of BCa in first degree relatives**					
None	711 (85.4)	498 (85.6)	1.00 reference	138 (80.2)	1.00 reference
1	110 (13.2)	76 (13.1)	1.02 (0.74–1.40)	32 (18.6)	1.47 (0.94–2.30)
≥2	12 (1.4)	8 (1.4)	1.23 (0.49–3.07)	2 (1.2)	0.90 (0.20–4.14)
			*p trend* = *0.74*		*p trend* = *0.18*
**Family history of BCa in mothers**					
No	711 (85.4)	498 (85.6)	1.00 reference	138 (80.2)	1.00 reference
Yes	69 (8.8)	45 (8.3)	0.95 (0.64–1.42)	16 (10.4)	1.21 (0.68–2.17)
Cancer >50 years	53 (6.8)	31 (5.0)	0.91 (0.56–1.48)	10 (5.5)	1.11 (0.54–2.26)
Cancer <50 years	15 (1.9)	14 (2.3)	1.22 (0.55–2.71)	6 (3.3)	2.24 (0.81–6.20)
**Family history of BCa in sisters**					
No	711 (85.4)	498 (85.6)	1.00 reference	138 (80.2)	1.00 reference
Yes	60 (7.8)	39 (7.3)	1.03 (0.67–1.58)	16 (10.4)	1.39 (0.76–2.54)
Cancer >50 years	32 (3.6)	21 (3.4)	0.87 (0.47–1.61)	5 (2.7)	0.82 (0.30–2.21)
Cancer <50 years	16 (1.8)	13 (2.1)	1.50 (0.69–3.26)	3 (1.6)	1.11 (0.31–4.02)
**Family history of BCa in daughters**					
No	711 (85.4)	498 (85.6)	1.00 reference	138 (80.2)	1.00 reference
Yes	2 (0.3)	7 (1.4)	**5.64 (1.16–27.5)**	4 (2.8)	9.25 (1.66–51.6)

^1^OR: Odds Ratios adjusted for age, ethnic origin, number of first-degree female relatives and famili history of prostate cancer in first-degree relatives.

^2^95% CI: 95% Confidence Interval.

We did not observed any association between PCa and family history of other cancer localizations in first-degree relatives (data not shown).

## DISCUSSION

The EPIdemiological study of Prostate CAncer (EPICAP) is a large population-based case-control study specifically carried out to investigate the role of environmental and genetic factors in prostate cancer. In concordance with previous studies, we showed that family history of PCa in first-degree relatives was associated with higher PCa risk [12–15].

We also showed a strong association between family history of BCa in first-degree relatives and PCa risk, particularly when BCa in first-degree relatives was before the age of 50. This association was also more pronounced for aggressive PCa. It has been previously shown that the relative risk for PCa was higher with four or more affected family members with any cancers [18]. On another way, the relationship between BCa and PCa have been already described in epidemiological studies based on BCa populations. In an Iceland cohort of women with BCa, an increased risk of PCa among relatives (particularly for first degree relatives) has been described [19]. Recently, findings suggest that PCa diagnosed among first-degree family members increases a woman's risk of developing BCa. However, different results were shown for male BCa and risk of PCa. [20, 21]. Those data and our findings data complement one another to assume a global familial risk of BCa and PCa.

Considering the cause of that segregation, many hypotheses have been suggested for those hormone-related cancers. Highly penetrance gene was suspected for this linkage. For PCa and ovarian cancer, a BRCA2 mutation accounts for most of the familiarity observed in families of BCa patients [19]. *BRCA2* is a tumor suppressor gene. It helps repair DNA double strand damage and, therefore, play a role in ensuring the stability of the cell's genetic material. Specific inherited mutations in *BRCA2* increase the risk of female breast and ovarian cancers. It has been also associated with increased risks of several additional types of cancer like PCa. Six percent of men with castration-resistant PCa could have pathogenic germline mutations of BRCA2 [22]. Our finding suggests a possible role of altered genes that could increase risk of cancers by common mechanisms like DNA repair leading to aggressive cancer in accordance with the linkage for PCa with higher Gleason score we founded [23]. Epidemiological studies indicate that dominantly inherited susceptibility genes with high penetrance may cause up to 5% to 10% of all PCa cases. Looking at common high penetrance alleles, several linkage studies for PCa have been completed, often with conflicting results [24, 25]. Then, using NGS in large studies with thousands of men, *HOXB13* -variant Gly84Glu was a validated gene associated with PCa [26]. However, this gene seems not to be implicated in BCa. Finally, common genetics variants, in genome wide association studies (GWAS), led to determine at least 100 SNP associated with a part of PCa risk [10]. It is also possible that those variants could have an impact on BCa.

Shared environmental risk factors could also contribute to the risks for cancers, even for cancer that could be driven by same genetic predisposition. The role of endocrine disruptors in hormone-related cancers (breast, prostate, testicular) is currently studied and could be another hypothesis to explain this association [27, 28].

Our results are based on a large population-based case-control study carefully designed to assess the role of environmental and genetic factors in prostate cancer occurrence. We were able to confirm that we identified all eligible cases over the study period by a posteriori cross-checking of the identified list of eligible cases with that of the Hérault cancer registry. Moreover, even though participation rate in cases was 75%, the age distribution and the Gleason score were comparable to those of the Hérault Cancer Registry for the years 2009–2011 (private communication) which indicates that cases included in the study were representative of all eligible cases. Controls were randomly selected from the general population of the département of Hérault using quotas on age (5-years). In addition, we established quotas by socioeconomic status (SES) to yield the control group similar to the general population of the département of Hérault of the same age in terms of SES. We compared the distribution by SES between our control group and the male general population of the *département* of Hérault and found no significant difference, indicating that no major selection bias by SES had occurred.

To minimize recall and misclassification bias, data were collected by a trained research clinical nurse using a standardized questionnaire. Cases and controls were asked to describe all their first-degree family members before asking if one of them had had a history of cancer. Our results confirmed the strong and known association between family history of prostate cancer in first-degree relatives and prostate cancer risk which give us some confidence about the relevance about the family history data that has been collected.

The number of first-degree relatives was statistically different between cases and controls with more relatives in controls than in cases (*p* = 0.026). This may have underestimate our results rather than explain them. However, all analyses have been adjusted for the number of relatives, limiting possible confusion bias.

In summary, a family history of early BCa in first degree may increase the risk of PCa, with possibly higher GS. BCa in daughter are related with the highest risk of PCa. These findings that is necessary to confirm in an independent study, may have significant implications according the role of inherited predisposition and shared environments causes of both cancers. As the contribution of a family history of BCa to PCa risk among relatives, and vice versa, has been shown, information risk for patients with family history of cancer, even if cases are described in relatives with opposite sex, is important. It pleads for wider proposals at genetic counselling and surveillance in patients with family history of cancer and particularly of BCa/PCa.

## MATERIALS AND METHODS

The “EPIdemiology Prostate CAncer” (EPICAP) study has been specifically designed to investigate the role of environmental and genetic factors in PCa.

### Case-control selection

EPICAP is a population-based case-control study carried out in the département of Hérault, a well delimited geographic area in the South of France. Details of the EPICAP objectives and study design have been previously described elsewhere [29].

Briefly, eligible cases were all newly diagnosed PCa cases in 2012–2013 in men under the age of 75 years old and residing in the département of Hérault at the time of diagnosis. All cases have been histologically confirmed.

The eligible controls were men randomly selected in the general population of Hérault, residing in the département of Hérault, who did not declare a PCa when they were included in the study and frequency matched to the cases by 5-year age group. Quotas on socioeconomic status have been defined to yield the control group comparable to the population of Hérault of the same age.

Overall, among the 1,098 eligible cases and the 1,109 eligible controls, 819 cases and 879 controls have been included in the study with a participation rate of 75% and 79%, respectively.

### Data collection

Cases and controls were face-to-face interviewed by an experienced research clinical nurses especially trained for this study, using a standardized computerized questionnaire that gathered detailed information on socioeconomic characteristics, personal medical history, family history of cancer, lifestyle, residential and occupational history.

Detailed information on family history of cancer in first-degree relatives (parents, brothers and sisters, children) was collected. First, we gathered information for each first-degree relative, particularly first name, year of birth and vital status. Second, cases and controls were asked, for each relative they describe, whether they may have had a cancer. In case of an affected relative, the number of cancer, the localization of each cancer based on 21 proposed localizations (leukemia, mouth, UADT, lung, oesophagus, stomach, liver, colorectal, breast, thyroid, skin, melanoma, bone, kidney, bladder, endometrial or ovarian, prostate, central nervous system, myeloma, Hodgkin disease, non-Hodgkin lymphoma, other), and the age of occurrence of each cancer was collected.

Specific medical data on prostate cancer cases were collected by the clinical research nurses from the medical records at the time of diagnosis and completed by the Hérault cancer registry (PSA value, Gleason Score, and TNM stage at diagnosis).

### Statistical analysis

All analyses were performed using SAS 9.4 (SAS Inc., Cary, North Carolina).

Odds ratios (OR) and their 95% confidence intervals (95% CI) were estimated using an unconditional logistic regression to investigate associations between family history of BCa in first-degree relatives and PCa risk. All analyzes were systematically adjusted for age, ethnicity and family history of PCa. We also adjusted analyses for potential confounding factors such as educational level or body mass index. The family structure (number of first-degree relatives) was also taken into account in the analyses.

All analyses were performed taking into account the aggressiveness of the tumor based on the Gleason score at diagnosis (low aggressiveness and intermediate: Gleason score < 7 and Gleason score = 7 including subjects for whom the two most commonly represented grades in the tumor are 3 + 4, as well as those for which the two grades are not known , high aggressiveness: Gleason score >7 and Gleason score = 7 including subjects for whom the sum of the two most frequently represented grades in the tumor are 4 + 3). Analyses were also stratified on family history of PCa in first degree relatives.

## Abbreviations

BCa: breast cancer
GS: Gleason score
OR: Odd ratio
PCa: prostate cancer
